# Near Infrared Spectroscopy Based Clinical Algorithm Applicability During Spinal Neurosurgery and Postoperative Cognitive Disturbances

**DOI:** 10.3390/medicina55050179

**Published:** 2019-05-21

**Authors:** Sniedze Murniece, Martin Soehle, Indulis Vanags, Biruta Mamaja

**Affiliations:** 1 Department of Anesthesiology, Riga East Clinical University Hospital, Hipokrata Street 2, LV1038 Riga, Latvia; biruta.mamaja@aslimnica.lv; 2 Department of doctoral studies, Riga Stradins University, Dzirciema Street 16, LV1007 Riga, Latvia; Indulis.Vanags@rsu.lv; 3 Department of Anesthesiology, University Hospital of Bonn, Sigmund-Freud Str.25, 53105 Bonn, Germany; Martin.Soehle@ukbonn.de; 4 Department of Anesthesiology, Paul Stradins Clinical University Hospital, Pilsonu Street 13, LV1002 Riga, Latvia

**Keywords:** spinal surgery, near-infrared spectroscopy (NIRS), regional cerebral oxygen saturation (rScO2), NIRS-based clinical algorithm, postoperative cognitive disturbances (POCD)

## Abstract

*Background and Objectives:* Postoperative cognitive disturbances (POCD) can significantly alter postoperative recovery. Inadequate intraoperative cerebral oxygen supply is one of the inciting causes of POCD. Near-infrared spectroscopy (NIRS) devices monitor cerebral oxygen saturation continuously and can help to guide intraoperative patient management. The aim of the study was to evaluate the applicability of the NIRS-based clinical algorithm during spinal neurosurgery and to find out whether it can influence postoperative cognitive performance. *Materials and Methods*: Thirty four patients scheduled for spinal neurosurgery were randomized into a study group (n = 23) and a control group (n = 11). We monitored regional cerebral oxygen saturation (rScO2) throughout surgery, using a NIRS device (INVOS 4100). If rScO2 dropped bilaterally or unilaterally by more than 20% from baseline values, or under an absolute value of 50%, the NIRS-based algorithm was initiated in the study group. In the control group, rScO2 was monitored blindly. To evaluate cognitive function, Montreal-Cognitive Assessment (MoCA) scale was used in both groups before and after the surgery. *Results*: In the study group, rScO2 dropped below the threshold in three patients and the NIRS-based algorithm was activated. Firstly, we verified correct positioning of the head; secondly, we increased mean systemic arterial pressure in the three patients by injecting repeated intravenous bolus doses of Ephedrine, ultimately resulting in an rScO2 increase above the approved threshold level. None of the three patients showed POCD. In the control group, one patient showed a drop in rScO2 of 34% from baseline and presented with a POCD. RScO2 drop occurred with other stable intraoperative measurements. *Conclusions*: A significant rScO2 drop may occur during spinal surgery in prone position despite other intraoperative measurements remaining stable, allowing it to stay otherwise unrecognized. Use of the NIRS-based clinical algorithm can help to avoid POCD in patients after spinal surgery.

## 1. Introduction

A significant number of patients of all ages present with postoperative decline in cognitive functions after non-cardiac surgery [1]. Postoperative cognitive disturbances (POCD) tends to improve over a certain period of time in the majority of patients [2], but some patients suffer from permanent cognitive impairment and other major and long-term consequences [3]. Cognitive disturbances following surgery are serious complications [4] that alter postoperative recovery significantly [5]. The exact etiology of the cerebral injury leading to POCD remains unclear, but assumingly includes a combination of patient-related, surgical, and anesthetic factors [6]. Many of these factors cannot be exactly identified. However, some of them can be modified, thereby minimizing the risk of POCD. Inadequate intraoperative cerebral blood flow and oxygen supply is one of the main causes of postoperative delirium and cognitive disturbances [7]. Appropriate oxygen delivery to the brain is one of the main tenets of anesthetic practice [8]. In spite of that, the brain remains one of the least monitored organs during surgery. 

In 1977, Jobsis [9] introduced near-infrared spectroscopy (NIRS) as a useful non-invasive tool for regional cerebral oxygen saturation (rScO2) monitoring, which provides continuous, real time information on the balance between cerebral oxygen delivery and consumption [5]. 

The technology of the NIRS cerebral oximeter depends on the transference and absorption of near-infrared light when it passes through tissue [10]. A cerebral oximeter unit consists of a monitor and two adhesive electrodes that are attached to patients’ forehead. The electrode incorporates a light source and light detectors. The light source radiates light in a near-infrared range of 650–940 nm that can penetrate the skull and the underlying brain tissue [11]. In the near-infrared wavelength spectrum, the main chromophores that can absorb light are hemoglobin and cytochrome c oxidase [10]. Based on the oxygenation status of the hemoglobin molecules, light spectrum changes occur [11]. Cerebral oximeter electrodes register the reflected light and quantify rScO2.

One of the intraoperative cerebral oxygen saturation monitoring benefits is the possibility to utilize a NIRS-based clinical algorithm [5] in cases in which rScO2 drops, and it aids in guiding the intraoperative patient management. The level of rScO2 values that should trigger initiation of the algorithm differ among authors, although the majority support either a 20% unilateral or bilateral reduction, or an absolute decline below rScO2 of 50% from the patients’ individual baseline value obtained before induction of anesthesia [12]. 

Neurosurgical spinal operations performed in the prone position carry a specific risk of an altered cerebral blood perfusion, which might impair oxygen supply to the brain. Reduced stroke volume and cardiac index might occur in patients lying in the prone position. Increased intra-abdominal pressure due to direct pressure on the vena cava inferior, and increased intrathoracic pressure with decreased left ventricular compliance and filling, leads to hypotension and reduced end organ perfusion, including the brain [13]. Brain blood supply may also fall in response to iatrogenic hypotension, which is often required during spinal surgery in an attempt to diminish intraoperative bleeding. 

Research on postoperative cognitive dysfunction after various types of surgeries has gained increased popularity. Recent studies show that maintenance of adequate intraoperative cerebral oxygen saturation can help to avoid POCD that lead to long term consequences [14,15]. POCD and their risk factors in relation to spinal surgery remain unclear and need further clarification.

The aim of the present study was to investigate whether application of an algorithm based on NIRS during spinal neurosurgery influences postoperative cognitive performance.

## 2. Materials and Methods

This prospective observational study included 34 patients scheduled for spinal neurosurgery. Inclusion criteria were: Patients over 18 years old, spinal surgery performed in prone position. Exclusion criteria were: Emergency spinal surgery, patients with known cerebrovascular or psychiatric disease, previous stroke, inability to undertake preoperative cognitive evaluation. Medical Research Ethics Committee of Riga Stradins University approved the study protocol and informed consent form (Approval Nr. 85/29.12.2016).

Preoperative blood tests were taken from all patients in the neurosurgical or neurological ward to obtain hemoglobin (Hb) and hematocrit (Ht) levels. Normal Hb values range from 12–16 g/dL for women and 14–18 g/dL for men and Ht from 36–48% for women and 40–54% for men [16]. Prior to surgery, we induced anesthesia with fentanyl 0.1–0.2 mg, propofol 1–2 mg/kg and cisatracurium 0.2 mg/kg for endotracheal intubation and maintained with a continuous infusion of fentanyl 0.03–0.06 μg/kg/min, cisatracurium 0.06–0.1 mg/kg/h and sevoflurane at MAC 0.6–0.8. Initial tidal volume for mechanical ventilation was set to 8 mL/kg and FiO2 to 0.5. Ventilation was set to keep end-tidal carbon dioxide (EtCO2) in the range of 35 to 45 mmHg. Intraoperative values such as non-invasive mean arterial pressure (MAP) was determined every 5 min, while heart rate (HR), peripheral oxygen saturation (SpO2) and end tidal carbon dioxide (EtCO2) were documented in the study protocol as single time point measurements every 5 min. At the end of the surgery, we recorded intraoperative blood loss and the duration of operation.

**Figure 1:**
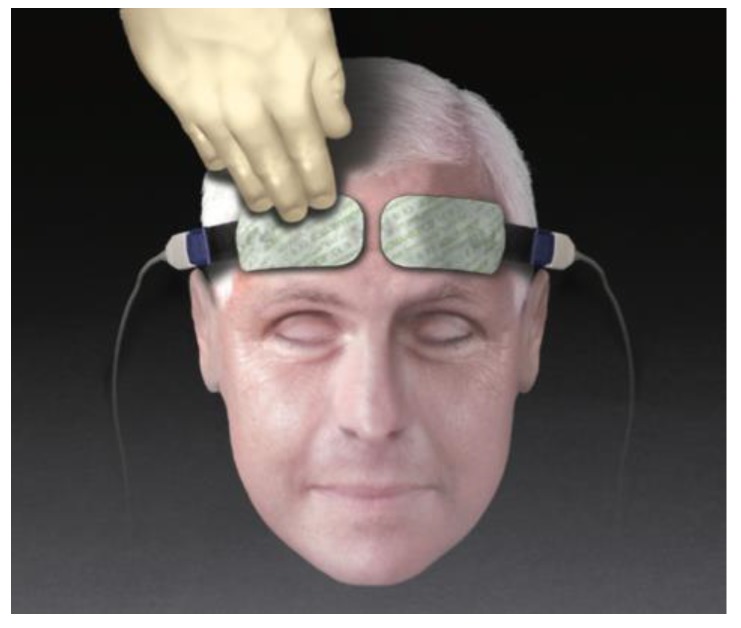
INVOS (IN Vivo Optical Spectroscopy) sensor placement on the patients’ forehead (picture taken from the INVOS Cerebral/Somatic Oximeter Operations Manual, Covidien 2010).

**Figure 2:**
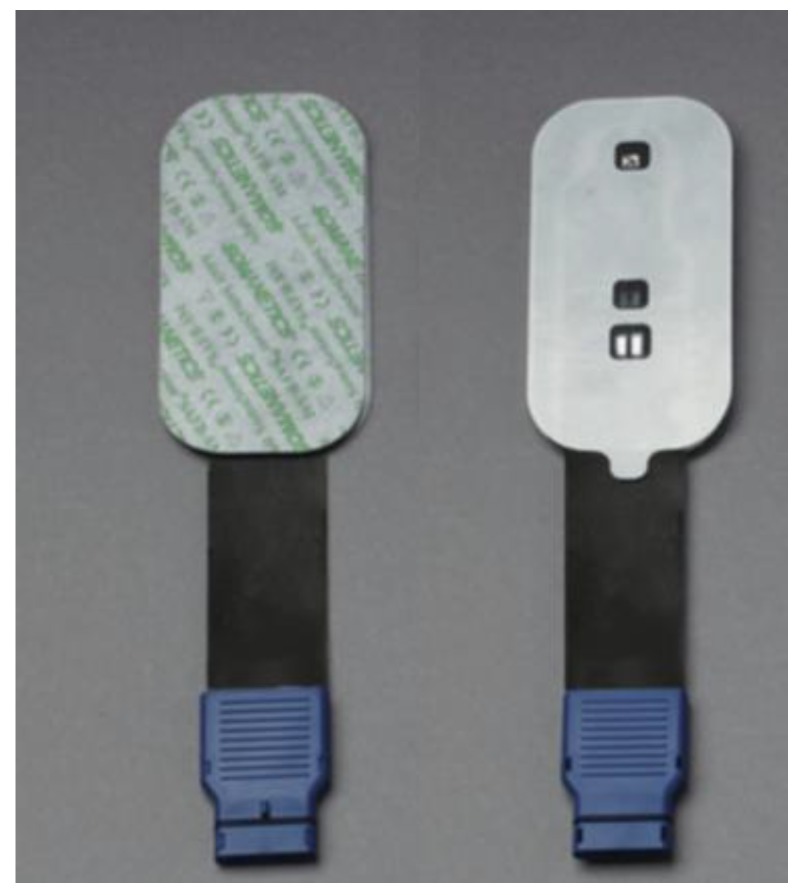
INVOS sensor (picture taken from The INVOS Cerebral/Somatic Oximeter Operations Manual, Covidien 2010).

**Figure 3:**
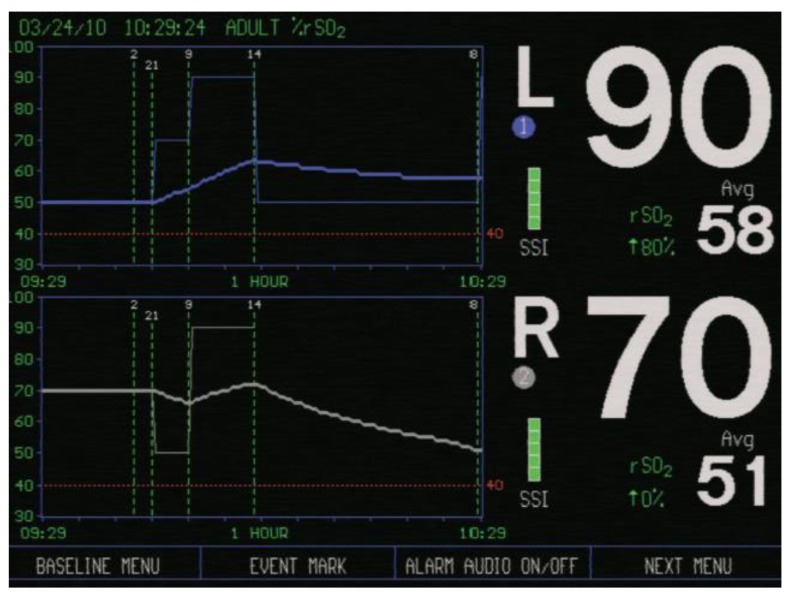
Screen of the INVOS cerebral oximeter showing regional cerebral oxygen saturation (rScO2) values above the left cerebral hemisphere (L) and right cerebral hemisphere (R) (picture taken from The INVOS Cerebral/Somatic Oximeter Operations Manual, Covidien 2010).

In all patients rScO2 was continuously monitored throughout the surgery, using NIRS device INVOS (IN Vivo Optical Spectroscopy) 4100 (Covidien, Minneapolis, MN, USA). The INVOS system utilizes near-infrared light at wavelengths that are absorbed by hemoglobin—730 and 810 nm. Two rScO2 sensors were placed on the patients’ forehead; one above the right (R) and one above the left (L) cerebral hemisphere after arriving in the operating room. One adhesive INVOS sensor pad was attached above the right eyebrow, the second above the left eyebrow (Figure 1). The distance between the light emitting diode (LED) and two INVOS sensor detectors was 3 and 4 cm (Figure 2). Sensors were connected to the INVOS monitor. The initial values on the screen were set as baseline rScO2 values. The patient is breathing room air before induction of anesthesia. By establishing baseline rScO2 values, INVOS further tracks the changes in rScO2 values for the left side and for the right side (Figure 3).

Patients were randomized into a study group (n = 23) and a control group (n = 11). In the study group intraoperative rScO2 values were kept in the range of 20% from baseline values or above an absolute rScO2 of 50%. As soon as rScO2 values dropped bilaterally or unilaterally under 20% from baseline values or under an absolute value of 50%, the NIRS-based clinical algorithm was initiated [5]. Based on the algorithm, steps were taken in the following order: The position of the head is verified (head’s rotation to the left/right side, and head’s flexion and extension is excluded) to rule out mechanical obstruction that could alter cerebral blood and oxygen supply; MAP is increased to maintain cerebral perfusion pressure; systemic oxygenation status is improved if arterial oxygen saturation (SaO2) is low; partial pressure of carbon dioxide (PaCO2) is normalized—hypocapnia or hypercapnia is treated; hemoglobin is optimized (according to the algorithm Hb less than 7–8 g/dL requires red blood cell transfusion); cardiac function is evaluated if the previous steps fail and have been ruled out; as a last step cerebral oxygen consumption should be estimated (convulsions, hyperthermia) [5]. As the algorithm was created for cardiac surgery patients, it also included a step in which central, aortic and superior vena cava catheters were inspected [5].

Control group patients received standard intraoperative anesthetic management, rScO2 was monitored blindly, and the investigator was unaware about the NIRS results. If MAP dropped below 65 mmHg [17], Ephedrin boluses were given, excessive bleeding was excluded. If SpO2 dropped under 94%, inspired oxygen concentration was raised above 50% [18]. If hemorrhage of over 500 ml occurred, arterial blood gas analyses were performed to detect Hb levels and to evaluate the necessity for a blood transfusion (transfusion trigger 7 to 9 g/dL during hemorrhage [19]).

To evaluate cognitive function we used the Montreal-Cognitive Assessment (MoCA) test in both groups before surgery and two days after the surgery to avoid intraoperatively-used anesthetic drug interaction with the test’s performance. The MoCA test evaluates the following parameters—attention, concentration, executive functions, memory, language, visuoconstructional skills, conceptual thinking, calculations and orientation [20]. The MoCA test is validated in different languages and is a screening test designed to detect mild cognitive dysfunction. The test takes 10–15 min. MoCA test scores range between 0 and 30 points. Postoperative cognitive decline was defined as reduction in postoperative MoCA test points compared to MoCA points before surgery.

Statistical analysis was performed using SPSS V.23. Groups were compared by *t*-test for parametric data and Mann-Whitney test for non-parametric data. Values were presented as mean ± standard deviation (SD). Statistical significance was assumed if *p* < 0.05. The receiver operating characteristic (ROC) curves, their respective 95% confidence intervals, and significance were compared between patients to identify predicting factors for POCD.

## 3. Results

Demographic characteristics (age, sex) and preoperative laboratory findings (Ht, Hb), duration of operation, intraoperative blood loss, intraoperative MAP, SpO2 and EtCO2 of the patients are shown in Table 1.

In the study and control groups, only mild changes were observed in mean rScO2 values before induction of anesthesia, during induction of anesthesia, in prone position during anesthesia, lying supine at the end of the anesthesia and surgery. The mean rScO2 values are shown in Table 2.

Considering all patients in the study group, an rScO2 drop below 20% from the patients’ individual baseline value measured prior to induction of anesthesia occurred in one patient (from rScO2 75% to 59%), and a drop below 50% occurred in 2 patients (from baseline rScO2 65% to 49% intraoperatively, and from baseline rScO2 62% to 47% intraoperatively). NIRS-based clinical algorithm was initiated and all the actions in the algorithm were taken, step by step.

As a first step, based on the NIRS-algorithm, the correct head position was verified—any flexion or rotation of the head was excluded, and a neutral head position was ensured. No increase in rScO2 values was observed. As a second step, MAP was raised. All three patients received boluses of Ephedrin (5–20 mg) resulting in an rScO2 increase above the threshold. No further interventions were necessary. Upon restoring non-critical rScO2, the algorithm was terminated. During the remaining surgery, rScO2 values remained stable and we observed no further decline below threshold. The lowest MAP of all three patients was 75–90 mmHg, recorded during the drop of rScO2. SpO2 and EtCO2 remained stable. No excessive hemorrhage was observed.

None of the three patients showed postoperative cognitive decline.

In the control group, the rScO2 of one of the patients (39 years old) dropped 34% below baseline values (from baseline rScO2 95% to lowest 63% intraoperatively). Other intraoperative readings: MAP 73–83 mmHg, SpO2 100% and EtCO2 35–37 mmHg remained stable during the decrease in rScO2, no excessive hemorrhage (150 mL). The patient showed postoperative cognitive decline (MoCA score decrease of 4 points).

Analyzing all patients included in the study, patients with intraoperative rScO2 drop below the threshold had a statistically significantly longer mean operation time (166 ± 79 min) compared to the patients who did not have significant rScO2 decrease (116 ± 34 min, *p* = 0.02).

In both the study and the control group, mean MoCA score did not change significantly before and after the surgery. In the study group, we noted a MoCA score of 24 ± 2 points before surgery and 25 ± 2 points after the surgery. Correspondingly, in the control group we noticed 26 ± 1 points and 25 ± 1 points before and after the surgery, respectively.

Analyzing all the patients of the study, 3 out of 23 study group patients and 4 out of 11 control group patients showed postoperative cognitive decline of 1–4 points. Regarding those patients we did not observe any statistically significant differences in preoperative laboratory values or intraoperative measurements as compared to the patients without cognitive decline. Medium preoperative and intraoperative rScO2 values did not differ significantly between the patients with postoperative cognitive decline and without.

**Figure 4:**
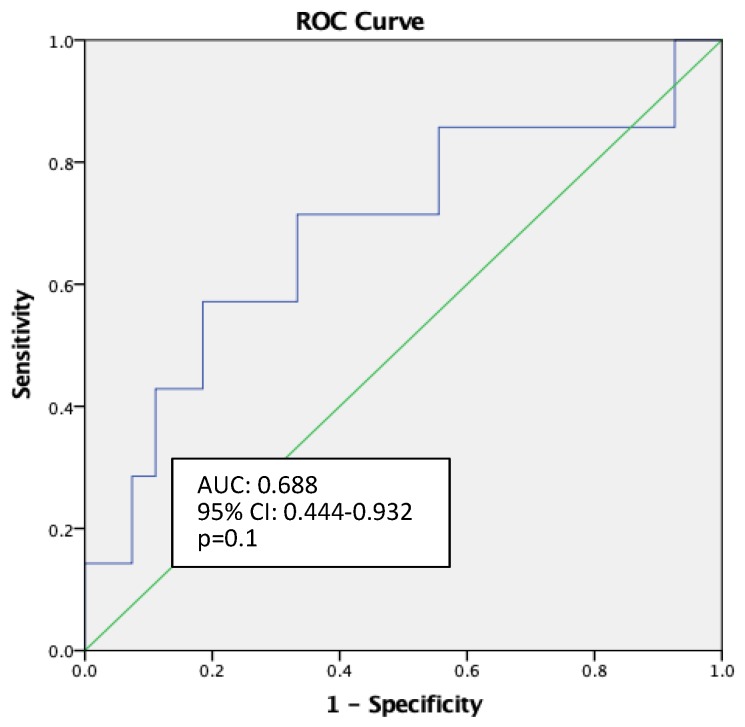
Receiver operating characteristic (ROC) curve for rScO2 monitoring as predictor of postoperative cognitive disturbances (POCD).

Receiver operating characteristic (ROC) curve analysis showed rScO2 monitoring to be a weak predictor of POCD (AUC: 0.688, 95% CI: 0.444–0.932, *p* = 0.1) (Figure 4). Neither did the other measurements (patient age, preoperative Hb, Ht level, duration of operation, intraoperative blood loss) appear to be strong predictors for postoperative cognitive decline, as assessed by ROC analysis.

## 4. Discussion

In our prospective study, NIRS-monitoring identified rScO2 drops of more than 20% from baseline and an absolute drop under 50% in 3 out of 23 study group patients and 1 out of 11 control group patients despite no significant changes in MAP, SpO2 and EtCO2. This leads to the assumption that standard minimum intraoperative monitoring used routinely during anesthesia, such as non-invasive blood pressure measurement, pulse oximeter, electrocardiogram, inspired and expired oxygen, and carbon dioxide [21], may not give sufficient information about brain oxygenation. In addition, it may convey a false impression that the concept of cerebral autoregulation and perfusion based on MAP works for every patient, thus adding a future argument in favor of NIRS-monitoring. Furthermore, study group patients, whose rScO2 decreased and the interventions required by the NIRS algorithm were made, did not show POCD, unlike one control group patient where significant rScO2 drop occurred, NIRS algorithm was not used and POCD was observed. Regarding our current study the depth of anesthesia was not monitored, thus representing a major limitation of the study since low BIS values and a prolonged period of deep anesthesia can emerge as risk factors for POCD [22]. Yet some studies are showing no correlation between depth of anesthesia and POCD [23].

A majority of studies show a positive correlation between lower intraoperative rScO2 values and POCD in older patients [24,25]. Our control group patient with intraoperative rScO2 decrease and postoperative cognitive decline was 39 years old, without any other known cardiovascular, neurological or other diseases. Therefore, younger patients with no comorbidities may suffer from POCD.

Shim et al. [4] performed a meta-analysis regarding risk factors for delirium after spinal surgery. After analyzing 33 articles the following risk factors indicated a significant association with postoperative delirium: Age (>65 years), female sex, number of medications used preoperatively, low preoperative hematocrit, albumin, duration of surgery and intraoperative blood loss [4]. In our study ROC curve analysis did not show patient age, preoperative Hb, Ht level, duration of operation or intraoperative blood loss to be strong predictors for postoperative cognitive decline. Nevertheless, more patients should be included in the study in order to estimate precise conclusions about factors that lead to POCD.

Regarding the monitoring of cerebral oxygen saturation during spinal surgery in prone position, only a limited number of studies are available. One of these studies suggests that patients undergoing surgery in prone position suffer from mild cerebral desaturation (rScO2 under 65%) 2.3 times more often (*p* = 0.009) as compared with patients that have been operated upon while lying supine [25], although the study included only elderly patients (≥68 years) [5]. Andersen et al. investigated the position of the head (neutral, head turned to left/right, head lifted from the head support, head down) and its influence on cerebral oxygenation in 48 spinal surgery patients [26]. As a result, they recommend a neutral head position, since a decline in rScO2 of 10 or more units was observed in rotated and lifted position of the head [26]. The verification of the position of the head also appears to be the first step in the NIRS-based clinical algorithm.

Postoperative screening of cognitive disturbances is not performed routinely, unless the patient presents with delirium with clearly visible clinical symptoms. Therefore, postoperative cognitive decline can stay unrecognized even at hospital discharge [14]. One of the tenets in our study was to employ a simple test, such as MoCA. The MoCA assessment requires just 10–15 min to evaluate the patients’ cognitive function. An additional benefit includes the relatively easy execution, which can be carried out by anesthesiologists or any other physician. This in turn increases the early detection of patients at risk of experiencing POCD. MoCA has shown a high sensitivity and specificity as a screening test for mild cognitive impairment, as well as for patients that have shown normal results during Mini-Mental State Examination (MMSE) [27,28,29].

Postoperative cognitive dysfunction or decline is a well-known clinical phenomenon with multifactorial origin [30,31], where intraoperative cerebral hypoxia is supposed to be one of the causes and, unlike other factors, can be modified. Non-invasive cerebral oxygenation monitoring including an easy to use monitor with an NIRS-based clinical algorithm can help to prevent cerebral desaturation intraoperatively and avoid hypoxic brain injury. The NIRS algorithm has been developed as a guideline for cases where cerebral desaturation occurs as it provides step by step interventions in a certain order which are specifically aiming to restore brain oxygenation [5]. Although, it is advisable to act only after careful evaluation of all available information of the patient, surgery and anesthesia, and after ruling out false-positive measurements due to NIRS device adhesive patch displacement [5].

A tailored patient approach, including innovative information and technologies to overcome patient-related health issues, has made the patient management more efficient. Many studies have already shown a clear benefit of intraoperative regional cerebral oxygenation monitoring [15,24,25,26,32] in different types of surgery. However, the question as to whether NIRS devices should be used routinely in all patients during spinal neurosurgery in prone position, or just for a specific group of patients, remains unclear.

## 5. Conclusions

A significant rScO2 drop for more than 20% from baseline value or below an absolute value of 50% rScO2 may occur during spinal surgery in prone position with stable intraoperative measurements (MAP, SpO2, HR, EtCO2), which would otherwise remain unnoticed.

The use of an NIRS-based algorithm in patients where the rScO2 drops for more than 20% from baseline values or under an absolute value of 50% can potentially help to avoid postoperative cognitive disturbances after spinal surgery.

## Figures and Tables

**Table 1:** Patients’ demographic characteristics (age, sex) and preoperative laboratory findings (Hb, Ht), duration of operation, intraoperative blood loss, intraoperative MAP, SpO2, EtCO2 in the study group and the control group.

	Study Group	Control Group	*p*-Value
Patients (n), sex (male/female)	23 (12/11)	11 (6/5)	
Age (years)	55 ± 14	58 ± 14	0.4
Hb level (g/dL)	13 ± 1	13 ± 1	0.1
Ht (%)	40 ± 5	38 ± 6	0.1
Duration of operation (min)	114 ± 45	130 ± 57	0.7
Blood loss (mL)	285 ± 287	345 ± 190	0.2
Intraoperative MAP (mmHg)	87 ± 13	80 ± 6	0.9
Intraoperative SpO2 (%)	99 ± 0.3	99 ± 1	0.7
Intraoperative EtCO2 (mmHg)	35 ± 1	34 ± 1	0.5

Values are shown as mean ± standard deviation. Hb—hemoglobin, Ht—hematocrit, MAP—mean arterial pressure, SpO2—peripheral oxygen saturation, EtCO2—end-tidal carbon dioxide.

**Table 2:** RScO2 in the study and control groups before induction of anesthesia, during induction of anesthesia, in prone position during anesthesia and lying supine at the end of the anesthesia and surgery.

Position	Cerebral Hemisphere	Mean rScO2 (%) in the Study Group	Mean rScO2 (%) in the Control Group	*p* Value
Before induction of anesthesia	Right	68 ± 7	68 ± 14	0.2
	Left	67 ± 9	73 ± 11	0.1
During induction of anesthesia	Right	74 ± 9	70 ± 13	0.4
	Left	73 ± 9	70 ± 10	0.4
Prone position during anesthesia	Right	73 ± 6	70 ± 6	0.3
	Left	73 ± 6	72 ± 4	0.7
Supine at the end of the anesthesia and surgery	Right	72 ± 8	70 ± 13	0.3
	Left	71 ± 8	70 ± 6	0.4

Values are shown as mean ± standard deviation. rScO2—regional cerebral oxygen saturation.
